# National Cohort of Compassionate Use of Meropenem–Vaborbactam: No Benefit over Meropenem for *Pseudomonas aeruginosa*

**DOI:** 10.3390/antibiotics13121152

**Published:** 2024-12-01

**Authors:** Aurélien Dinh, Alexandre Bleibtreu, Clara Duran, Frédérique Bouchand, Alexie Bosch, Jullien Crozon-Clauzel, Mariam Roncato-Saberan, Morgan Matt, André Boibieux, Annlyse Fanton, Heidi Wille, Elise Fiaux, Benoît Pilmis, Marie Lacoste, Quentin Saint-Genis, Caroline Thumerelle, Patricia Pavese, Fanny Vuotto, Eric Senneville, Anaïs Potron, Stéphane Corvec, David Boutoille, Katy Jeannot, Laurent Dortet, on behalf of the Meropenem-Vaborbactam French Study Group

**Affiliations:** 1 Infectious Disease Unit, Raymond-Poincaré University Hospital, AP-HP Paris Saclay University, 92380 Garches, France; clara.duran@aphp.fr; 2 Infectious Disease Unit, La Pitié-Salpétrière University Hospital, AP-HP University of Paris, 75013 Paris, France; alexandre.bleibtreu@aphp.fr; 3 Pharmacy, Raymond-Poincaré University Hospital, AP-HP Paris Saclay University, 92380 Garches, France; frederique.bouchand@aphp.fr; 4 Infectious Disease, Chambery Hospital, 73000 Chambery, France; alexie.bosch@ch-metropole-savoie.fr; 5 Surgical Intensive Care Unit, University Hospital, 69000 Lyon, France; jullien.crozon-clauzel@chu-lyon.fr; 6 Infectious Disease Unit, 17000 La Rochelle, France; mariam.roncato-saberan@ght-atlantique17.fr; 7 Infectious Disease Unit, Private Hospital Bordeaux Nord Aquitaine, 33300 Bordeaux, France; dr.mmatt@gbna-sante.fr; 8 Infectious Disease Unit, University Hospital, 69000 Lyon, France; andre.boibieux@chu-lyon.fr; 9 Pulmonology Department, University Hospital, 21231 Dijon, France; annlyse.fanton@chu-dijon.fr; 10 Infectious Disease Department, Centre hospitalier de la Côte Basque, 64100 Bayonne, France; hwille@ch-cotebasque.fr; 11 Infectious Disease Department, University Hospital, 76000 Rouen, France; elise.fiaux@chu-rouen.fr; 12 Infectious Disease Unit, Hopital Saint Joseph, 75014 Paris, France; bpilmis@ghpsj.fr; 13 Infectious Disease Department, Alpes Leman Hospital, 74130 Contamine Sur Arve, France; mlacoste@ch-alpes-leman.fr; 14 Surgical Intensive Care Unit, University Hospital, 86000 Poitiers, France; saint-genis@sfr.fr; 15 Pediatric Pulmonology Department, University Hospital, University Lille, CHU Lille, 59000 Lille, France; caroline.thumerelle@chu-lille.fr; 16 Infectious Disease Department, University Hospital, 38000 Grenoble, France; ppavese@chu-grenoble.fr; 17 Infectious Disease Department, University Hospital, 59000 Lille, France; fanny.vuotto@chu-lille.fr; 18 Infectious Disease Department, University Hospital, 59599 Tourcoing, France; senneric670@gmail.com; 19 Microbiology Laboratory, University Hospital, 25000 Besançon, France; anais.potron@univ-fcomte.fr (A.P.); katy.jeannot@univ-fcomte.fr (K.J.); 20 Microbiology Laboratory, CHU Nantes, University Nantes, INCIT U1302, 44000 Nantes, France; stephane.corvec@chu-nantes.fr; 21 Infectious Disease Department, CIC 1413 INSERM, University Hospital, 44000 Nantes, France; david.boutoille@chu-nantes.fr; 22 Microbiology Laboratory, Bicêtre University Hospital, AP-HP Paris Saclay University, 94270 Le Kremlin-Bicêtre, France; laurent.dortet@aphp.fr; 23 Associate French National Center for Antimicrobial Resistance, 94270 Le Kremlin-Bicêtre, France

**Keywords:** meropenem–vaborbactam, bacterial resistance, carbapenem, respiratory tract infection, *Pseudomonas aeruginosa*

## Abstract

Background: Meropenem–vaborbactam (MEM-VAB) is a novel carbapenem-beta-lactamase-inhibitor combination that demonstrates activity against carbapenem-resistant (CR) Gram-negative bacteria, and more specifically KPC-producers, since vaborbactam is an effective inhibitor of KPC enzymes in vitro. This study aimed to describe the initial uses and efficacy of MEM-VAB for compassionate treatment during the first 21 months following its early access in France. Method: A national multicenter retrospective study was conducted, including all patients who received at least one dose of MEM-VAB between 20 July 2020, and 5 April 2022. Clinical characteristics and outcomes were collected using a standardized questionnaire. The minimum inhibitory concentration (MIC) of antimicrobials, and complete genome sequencing of bacteria were performed when bacterial isolates were available. Results: Ultimately, 21 patients from 15 French hospitals were included in the study. The main indication for MEM-VAB treatment was respiratory tract infections (*n* = 9). The targeted bacteria included *Pseudomonas aeruginosa* (*n* = 12), *Klebsiella pneumoniae* (*n* = 3), *Enterobacter spp* (*n* = 3), *Citrobacter freundii* (*n* = 1), *Escherichia coli* (*n* = 1), and *Burkholderia multivorans* (*n* = 1). Overall, no significant advantage of vaborbactam over meropenem alone was observed across all strains of *P. aeruginosa* in terms of in vitro susceptibility. However, MEM-VAB demonstrated a notable impact, compared to carbapenem alone, on the MIC for the two KPC-3-producing *K. pneumoniae* and *B. multivorans*. Conclusions: MEM-VAB seems effective as a salvage treatment in compassionate use, but vaborbactam was shown to lack benefits compared to meropenem in treating *P. aeruginosa*-related infections. Therefore, it is crucial to compare meropenem to MEM-VAB MICs, particularly for *P. aeruginosa*, before prescribing MEM-VAB.

## 1. Introduction

Carbapenem resistance in Gram-negative bacteria represents a significant global threat, leading to increased mortality rates, particularly among vulnerable patient populations [1,2].

Meropenem–vaborbactam (MEM-VAB) is a novel antibiotic that exhibits in vitro activity against multidrug-resistant (MDR) Gram-negative pathogens [3]. It has been approved to treat hospital-acquired pneumonia (HAP), including ventilator-associated pneumonia (VAP), in Europe, in addition to the treatment of complicated intra-abdominal and urinary tract infections and acute pyelonephritis. MEM-VAB was also approved for bacteremia that occurs in association with any of these infections and for infections due to aerobic Gram-negative organisms, where treatment options are limited [4].

Regarding Gram-negative organisms where treatment options are limited, carbapenem-resistant (CR) Enterobacterales or *P. aeruginosa* are particularly concerning. In both cases, CR might involve the production of a carbapenemase or result from a combination of decreased outer-membrane permeability and the overproduction of a β-lactamase with expanded spectrum activity, such as Ambler’s class A extended-spectrum β-lactamases (ESBL) and Ambler’s class C cephalosporinases (AmpC). The main carbapemases encountered in Enterobacterales and *P. aeruginosa* include (i) Ambler’s class A carbapenemase, such as KPC [5] and GES type, (ii) Ambler’s class B metallo-β-lactamases, including NDM, VIM and IMP types [6,7], and (iii) Ambler’s class D carbapenem-hydrolyzing enzymes of the OXA-type (OXA-48-like in Enterobacterales [8] and OXA-198/OXA-677 in *P. aeruginosa* [9,10].

Regarding its inhibitor activity towards ESBL and AmpC, vaborbactam has been demonstrated to be a relevant option for the treatment of infections caused by Carbapenem-Resistant Enterobacterales (CRE) that do not produce carbapenemase (CRE non-CPE (Carbapenemase-Producer Enterobacterales)) [11]. Although MEM-VAB has been reported to be less effective than ceftazidime–avibactam and imipenem–relebactam, the systematic testing of all β-lactam/β-lactamase inhibitors (imipenem–relebactam, ceftazidime–avibactam, and MEM-VAB) has proven to be a valuable strategy for CRE non-CPE infections. This approach is justified by the diversity of the resistance mechanisms involved, which makes it impossible to predict susceptibility to all β-lactam/β-lactamase inhibitors based on the testing of a single molecule [11]. However, despite imipenem–relebactam and MEM-VAB initially being proposed as possible treatments of infections caused by OXA-48-like-producing Enterobacterales, recent data clearly established that neither imipenem–relebactam nor MEM-VAB possess a relevant in vitro benefit compared to carbapenem alone [12].

The results of the phase 3 clinical trial (TANGO II) to evaluate the safety, efficacy, and tolerability of MEM-VAB monotherapy in treating patients with serious CRE infections versus the best available therapy (BAT) were very encouraging. While TANGO I showed a non-inferiority of MEM-VAB in comparison with piperacillin–tazobactam for treating cUTIs, TANGO II, indicated that this new combination was safer and more effective than the BAT for managing infections caused by CRE. Evidence on the real-life use of MEM-VAB is limited, but supports its efficacy and safety in the treatment of CRE infections [13,14,15].

However, there is a scarcity of real-life data regarding indications during the compassionate use and the clinical effectiveness of MEM-VAB. This study aims to evaluate the efficacy of MEM-VAB in a compassionate use setting during the early access program in France, and to define the targeted strains according to their molecular resistance gene background.

## 2. Results

Overall, the study included 21 patients from 15 French university hospitals (Table 1).

The mean age was 53.2 ± 21.0 years, with a sex ratio of 3.2. The main comorbidities observed were chronic renal failure (*n* = 7), chronic respiratory failure (*n* = 6), and neurological disease (*n* = 3). Nine patients were considered immunocompromised, primarily due to diabetes mellitus (*n* = 7) or being solid organ transplant recipients (*n* = 3).

At first administration of MEM-VAB, 15 and 5 patients were in medical and ICU settings, respectively. The main sites of infection were respiratory tract infections (*n* = 9), intra-abdominal infections (*n* = 6), urinary tract infections (*n* = 3), bloodstream infections (*n* = 3), and bone and joint infections (*n* = 2). Two patients had multi-site infections: vascular infection/bloodstream infections and intra-abdominal infections and vascular infections and respiratory tract infections. At the start of treatment, three patients presented with septic shock. The mean (SD) Sequential Organ Failure Assessment (SOFA) score was 8.7 (±9.6). MEM-VAB was used as the first-line therapy for 6 out of 21 patients.

Four patients benefited from an image-guided drainage and fifteen patients had previous antibiotic treatment.

The most commonly prescribed MEM-VAB regimen was 2 g three times a day (10 patients), while six patients received 2 g four times a day. Patients with acute renal failure required a dosage of 750 mg three times a day (4 patients) or twice a day (1 patient). Only one patient received 1 g once a day. The median duration of MEM-VAB treatment was 14 Days (IQR: 10.0–40.0).

MEM-VAB administration was an extended infusion (3–4 h) (20 patients) except for one patient receiving a 1 h infusion. Adverse events were reported, including neurological issues (*n* = 1), acute kidney injury (*n* = 1), and *Clostridioides difficile*-associated infection (*n* = 1).

Concomitant active antibiotic treatment on pathogen suspected was prescribed among seven patients: fluoroquinolones (*n* = 4) and colistin (*n* = 3)

The pathogens identified were *P. aeruginosa* (*n* = 12), *K. pneumoniae* (*n* = 3), *Enterobacter spp* (*n* = 3), *E. coli* (*n* = 1), *C. freundii* (*n* = 1), and *Burkholderia multivorans* (*n* = 1). In six patients, the infection was polymicrobial.

A total of 11 strains were collected in 11 patients and analyzed in the FNRC-AR (Table 2). The results of antimicrobial susceptibility testing, MLST, and antibacterial resistance genes are presented in Table 2.

Although all strains were initially categorized as resistant to meropenem (MIC > 8 mg/L) and susceptible to MEM-VAB (MIC ≤ 8 mg/L) by the local hospital laboratory, only five isolates were categorized as resistant to meropenem by the national FRNRC-AR. Additionally, two *P. aeruginosa* isolates were resistant to MEM-VAB. In all *P. aeruginosa* isolates (*n* = 5), the addition of vaborbactam to meropenem did not modify the MIC (Table 2), suggesting that vaborbactam did not provide any advantage in treating *P. aeruginosa* infections.

MEM-VAB was very effective against the two KPC-3-producing *K. pneumoniae* leading to the MIC of meropenem being lowered from 16 or 8 mg/L to ≤0.06 mg/L. A significant MIC reduction was also observed for the *B. multivorans* isolate with MEM-VAB MIC, at 4 mg/L compared to 16 mg/L for meropenem alone. Finally, as expected, the addition of vaborbactam did not have any effect on the NDM-1-producing *Enterobacter hormaechei* since vaborbactam is not an MBL inhibitor.

In total, 13 out of 21 patients met the predefined criteria for clinical cure, indicating an overall cure rate of 61.9%.

## 3. Methods

We conducted a comprehensive national retrospective study involving adult patients who received at least one dose of MEM-VAB between 20 July 2020, and 5 April 2022. Prescribers were provided with standardized questionnaires to collect baseline patient characteristics, infection types, management approaches, microbiological data, reasons for MEM-VAB use, treatment dosage and duration, adverse events, and outcomes. The research adhered to the principles outlined in the Declaration of Helsinki, as well as national and institutional standards. Patients were informed that MEM-VAB was administered as part of a compassionate use program, and their clinical data, after anonymization, would be used for research purposes.

Immunosuppression was defined as the presence of conditions such as diabetes melitus, asplenia, neutropenia, agammaglobulinemia, organ transplant, hematologic malignancies, HIV infection with a CD4 cell count <400/mm^3^, or end-stage liver disease. Immunosuppressive treatment was defined as the use of systemic corticosteroids at a daily dose >20 mg of prednisolone equivalent for at least three weeks, chemotherapy, or other immunosuppressive drugs (cyclophosphamide, azathioprine, cyclosporine, etc.).

The outcome was assessed by the investigators during the patients’ most recent visit after completing MEM-VAB treatment. Cure was defined as survival with no residual signs of infection and pathogen eradication.

Quantitative variables are presented as mean ± standard deviation (SD) or median and interquartile range (IQR), while qualitative variables are reported as the number of occurrences and relative frequencies. All statistical analyses were conducted using SPSS Statistics v.17.0 software (SPSS Inc., Chicago, IL, USA).

All available bacterial isolates were centralized at the French National Reference Center for Antimicrobial Resistance (FRNRC-AR) (Bicêtre University Hospital, Le Kremlin-Bicêtre, France). Antibiotic Minimal inhibitory Concentrations (MICs) were determined via broth microdilution (BMD) using customized Sensititre microplates (ThermoFisher, Waltham, MA, USA) according to the guidelines of the European Committee on Antimicrobial Susceptibility Testing (EUCAST) [16]. The clinical breakpoints used for interpretation were those defined by EUCAST 2024 [16]. All bacterial isolates underwent whole genome sequencing using Illumina technology, as described previously [17]. Antimicrobial resistance genes were identified using the Resfinder server v3.2 (http://genepi.food.dtu.dk/resfinder, accessed on 10 June 2020) and CARD database (https://card.mcmaster.ca, accessed on 10 June 2020). MultiLocus Sequence Typing (MLST) was performed using the MLST 2.0 server (http://www.genomicepidemiology.org/, accessed on 10 June 2020).

## 4. Discussion

Our study focused on the early use of MEM-VAB as a salvage treatment for multidrug-resistant Gram-negative pathogens within the compassionate use program in France. The primary indication for MEM-VAB administration was respiratory tract infections caused by *P. aeruginosa*.

Despite the challenging clinical scenario of treating immunocompromised patients with limited therapeutic options, our study demonstrated a cure rate of 13 out of 21 cases in the global population, including the five patients infected with a meropenem-resistant strain. These patients had significant comorbidities that are typically associated with high mortality rates and low success rates with conventional antibiotics. These findings highlight the urgent need for improved antibiotic agents like MEM-VAB to treat such complex infections.

MEM-VAB has demonstrated notable activity against KPC-producing Enterobacterales, which are among the most prevalent and globally reported CPE [18]. Previous studies have consistently shown a high rate of in vitro susceptibility of KPC-producing Enterobacterales to MEM-VAB, particularly when compared to other broad-spectrum antibiotics. Furthermore, the existing literature primarily focuses on the activity of MEM-VAB against KPC-producing Enterobacterales and reports favorable clinical outcomes [3]. In a large-scale study on the clinical use of MEM-VAB, Shields et al. found that MER-VAB was used as a first-line treatment option against the KPC producers. Twenty patients were included in the study. They presented with BSI, bacterial pneumonia including VABP, tracheobronchitis, SSTI (skin and soft tissue infection), cIAI, and UTI. The most commonly isolated pathogens were *K. pneumoniae* (*n* = 14), *Klebsiella oxytoca* (*n* = 2), and *E. coli* (*n* = 2). The overall clinical success rate and 30-day survival ratio were 65% and 90% [13].

Another multicenter, retrospective analysis of the clinical efficiency of MER-VAB was conducted in the USA in 2017–2020, including 126 patients infected by MDR GNB, including CRE pathogens (mainly *K. pneumoniae*, *E. coli*, and *Enterobacter* spp.). In addition, patients (*n* = 10) with confirmed non-CRE infections (*A. baumannii* and *P. aeruginosa*) were included in the study population. The most prevalent infections were respiratory tract (38.1%), abdominal cavity (19.0%), and urinary tract (13.5%). The mortality rate on day 30 was 18.3% (23/126). The positive clinical efficacy of MER-VAB against *P. aeruginosa* (90-day mortality rate 1/8, recurrence of infection within 30 days to 2/8) is encouraging; however, the population of the study was not sufficiently large to draw clear conclusions [15].

The emergence of carbapenemase-producing Enterobacterales has driven the development of new antibiotics, mostly beta-lactams combined with beta-lactamase inhibitors, such as MEM-VAB and ceftazidime–avibactam. However, few comparative studies between these new agents have been performed.

A multicenter retrospective cohort study involving 131 adults with CRE infections, primarily *Klebsiella pneumoniae* carbapenemase (KPC)-producers, compared outcomes in patients treated with ceftazidime–avibactam (*n* = 105) and MEM-VAB (*n* = 26). The study found no statistically significant difference in clinical success rates between the two groups (62% versus 69%; *p*-value = 0.49). However, the development of resistance was observed more frequently among patients receiving ceftazidime–avibactam monotherapy [19].

In France, infections caused by KPC-producing bacteria remain rare, as supported by the FNRC-AR or the European Centre for Disease Prevention and Control (ECDC) data [20]. Surprisingly, in our study, we observed that the primary use of MEM-VAB was mostly directed against non-fermenting Gram-negative bacteria. The prevalence of carbapenem-resistant non-fermenters has now surpassed that of Enterobacterales in many healthcare settings, posing a significant challenge for managing severe infections [20,21]. Carbapenem-resistance mechanisms among non-fermenting Gram-negative bacteria involve both transmissible factors such as carbapenemase production, as well as intrinsic resistance mechanisms including a combination of porin loss and efflux pumps. Here, we demonstrate that the in vitro efficacy of MEM-VAB against *P. aeruginosa* is comparable to that of meropenem alone. The preference for using MEM-VAB rather than meropenem specifically against *P. aeruginosa* may be attributed to the availability of MEM-VAB MIC gradient strips to efficiently test MEM-VAB susceptibility during this first phase of early access of the molecule. Accordingly, it might be possible that developments in microbiology could allow for MEM-VAB to be tested using these MIC gradient strips without the need to also test meropenem to determine any increase in vaborbactam. Accordingly, our results highlight that it is crucial to perform an accurate susceptibility testing of meropenem against *P. aeruginosa*, as the addition of vaborbactam does not confer any microbiological advantage or result in a lower MIC.

Different studies have reported limited advantages of MEM-VAB compared to the use of meropenem alone for the treatment of infections caused by *Pseudomonas aeruginosa*, except in rare cases [22,23,24,25,26].

Based on our data, the most effective in vitro options for treating difficult-to-treat resistant *P. aeruginosa* strains are still colistin and ceftolozane–tazobactam.

However, in the case of the two KPC-producing *K. pneumoniae* and the *B. multivorans* isolates, the use of MEM-VAB resulted in a significant reduction in MIC compared to meropenem alone. In both cases, the vaborbactam efficiently inhibited the Ambler class A carbapenemase (KPC-3 for *K. pneumoniae* isolates and the intrinsic PenA for *B. multivorans*). Of note, the same kind of efficient inhibition was observed with relebactam (Table 2). However, in these three cases, the MEM-VAB MIC was lower than the results obtained using imipenem–relebactam treatment. These findings highlight the potential benefit of MEM-VAB in treating infections caused by these specific resistant strains. Nevertheless, depending on which molecular mechanisms (genotype) are involved in the phenotypic resistance pattern (phenotype), it would be interesting to assess the current and future β-lactam/β-lactamase-inhibitor associations. MEM-VAB remains an excellent therapeutic option with low MICs in cases of KPC-producing Enterobacterales, as observed in our study. However, for multi-drug-resistant *P. aeruginosa* strains, imipenem–relebactam, cefepime–enmetabactam, cefepime–zidebactam, cetolozane–tazobactam, eravacycline, or cefiderocol should be evaluated depending on the genetic background [27,28,29].

Regarding the CRE non-CPE isolates, our results confirm the previously described potential efficacy of any β-lactam/β-lactamase inhibitor association [11] and the need to test all of them without preconceptions to identify the best therapeutic option.

Finally, the strengths of our study lie in its real-world design, which reflects actual clinical needs and off-label use, in contrast to randomized controlled trials. Moreover, it highlights the safety profile of MEM-VAB. Both study types are complementary and can contribute to a more comprehensive understanding of the effectiveness and appropriate use of novel antibiotic treatments. The main limitations of our study stem from its retrospective design, which may lead to potential missing data and the biases inherent to this methodology. Moreover, the database does not provide information on the rationale behind the choice of MEM-VAB over other antibiotics. This decision was likely influenced by the availability of susceptibility testing for MEM-VAB. Notably, all strains remained susceptible to cefiderocol, and most were susceptible to ceftazidime–avibactam.

## 5. Conclusions

Our study evaluated the efficacy of MEM-VAB during the early access program in France, demonstrating a high cure rate, particularly against targeted strains, notably *Klebsiella pneumoniae* carbapenemase (KPC)-producing strains. However, MEM-VAB was predominantly prescribed for the treatment of respiratory tract infections caused by *Pseudomonas aeruginosa*. Our results suggest that MEM-VAB does not offer a significant advantage over meropenem alone in the management of *P. aeruginosa* infections.

It is crucial to emphasize the importance of conducting accurate susceptibility testing for β-lactam alone in parallel with β-lactam/β-lactamase-inhibitor associations before considering β-lactam/β-lactamase inhibitor prescriptions.

These findings highlight the need for a comprehensive evaluation of treatment options, taking into account both the susceptibility patterns of *P. aeruginosa* and the efficacy of different antibiotics, to provide the most effective and appropriate treatment for patients.

## Figures and Tables

**Table 1:** Characteristics of the population treated with meropenem–vaborbactam.

Patients (*n*)	21
Age (median, SD)	52.4 ± 21.2
Sex ratio (M/F)	3.20
Comorbidities (*n*, %)	
Chronic renal failure	7 (33.3)
Chronic respiratory failure	6 (28.6)
Heart disease	3 (14.3)
Neurological disease	3 (14.3)
Liver disease	2 (9.5)
Immunosuppression	9 (42.9)
Diabetes mellitus	7 (33.3)
Solid organ transplant	3 (14.3)
Immunosuppressive treatment	3 (14.3)
Neutropenia (<500/µL)	1 (4.8)
Chemotherapy	1 (4.8)
Corticosteroid therapy (>20 mg/d)	1 (4.8)
Antibiotic allergy (*n*, %)	4 (19.0)
Renal clearance (mL/min) (mean, SD)	139.1 ± 99.8
Primary site of infection (n, %)	
Respiratory tract infection	9 (42.9)
Intra-abdominal infection	5 (23.8)
Urinary tract infection	3 (14.3)
Bacteremia	3 (14.3)
Bone and joint infection	3 (14.3)
Severity (*n*, %)	
SOFA score (mean, SD)	8.7 ± 9.6
Intensive care unit admission	6 (28.6)
Mechanical ventilation	6 (28.6)
Septic shock	3 (14.3)
Vasopressor use	3 (14.3)
Fluid resuscitation	3 (14.3)
Biology at baseline (mean, SD)	
Leukocytosis (G/L)	11.5 ± 6.3
Hemoglobin (g/dL)	10.7 ± 2.6
Neutrophils (G/L)	9.3 ± 6.0
C-reactive protein (CRP) (mg/L)	79.1 ± 61.9
Number of antibiotic lines (median, IQR)	1.0 (1.0–2.0)
Meropenem–vaborbactam treatment duration (days, median, IQR)	14 (10.0–40.0)
Microbiology (*n*, %)	
*Pseudomonas aeruginosa*	12 (52.4%)
*Enterobacter* spp.	3 (14.3)
*Klebsiella pneumoniae*	3 (14.3)
*Escherichia coli*	1 (4.8)
*Citrobacter* spp.	1 (4.8)
*Burkholderia multivorans*	1 (4.8)
Bacteria resistance (*n*, %)	
Carbapenem-resistant without carbapenemase production	3 (14.3)
KPC	2 (9.5)
VIM	2 (9.5)
NDM	1 (4.8)
OXA-48	1 (4.8)
Outcome (n, %)	
Failure	7 (33.3)
Death due to infection	1 (4.8)
Suppressive antibiotic treatment	1 (4.8)

**Table 2:** Microbiology analyses.

Patient	1	2	3	4	5	6	7	8	9	10	11
Age	75	32	21	43	75	81	83	72	64	33	18
Sex	F	M	M	M	M	F	M	M	M	M	F
Infection	Bone and joint	Bone and joint	Respiratory tract	Intra-abdominal	Urinary tract	Intra-abdominal	Intra-abdominal	Vascular	Intra-abdominal Vascular	Respiratory tract	Respiratory tract
Bacteria	*K. pneumoniae*	*P. aeruginosa*	*Burkholderia multivorans*	*E. cloacae*	*P. aeruginosa*	*K. pneumoniae*	*P. aeruginosa*	*E. hormaechei*	*K. pneumoniae*	*P. aeruginosa*	*P. aeruginosa*
Subtype ST	ST-307	ST-2307		ST-873	ST-2659	ST-307	ST-968	ST-78	ST-147	ST-274	ST-139
Carbapenemase	KPC-3	No	PenA	VIM-4	No	KPC-3	No	NDM-1	No	No	No
Other beta-lactamases (acquired or intrinsic)	CTX-M-15, OXA-1, OXA-9, TEM-1, SHV-28	Altered OprD + OXA-494 + PDC-3		ACT-59 + TEM-1	hyper AmpC+ Altered OprD OXA-50 + PDC-3	OXA-9 + TEM-1 + SHV-28	Altered OprD + PDC-31 + OXA-1031	ACT-24	CTX-M-15, OXA-1, TEM-1, SHV-11	Altered OprD + PDC-330, OXA-486	Altered OprD + PDC-346, OXA-1026
Other genes of bacterial resistance (acquired or intrinsic)	aac(3)-IIe, aac(6′)-Ib-cr, aph(3″)-Ib, aph(6)-Id, catB3, dfrA14, oqxA, oqxB, qnrB1, sul2, vgaC, fosA	aph(3′)-IIb, catB7, fosA		aac(6′)-Ib, aac(6′)-II, aad2, ant(2″)-I1, aph(3″)-Ib, aph(6)-Id, dfrA1, fosA, mcr-9, oqxA, oqxB, qnrA1, sulI, tetA, vgaC	aph(3′)-IIb, catB7, fosA, crpP1	oqxA, oqxB, fosA	aph(3′)-IIb, catB7, fosA	aph(3′)-VI, dfrA15, qnrA1, sul1, oqxA, oqxB, fosA	aac(3)-IIe, aac(6′)-Ib-cr, oqxA, oqxB, sul2, fosA	aph(3′)-IIb, catB7, fosA	aph(3′)-IIb, catB7, fosA
MIC											
Aztreonam	>32	>32	>32	>32	16	>32	>32	32	>32	>32	>32
Colistin	≤0.5	4	>16	0.5	2	0.5	2	≤0.5	≤0.5	1	≤0.5
Imipenem	8	>8	>8	4	>8	16	8	2	2	>8	8
Cefepime	>16	>16	>16	16	8	>16	0.5	>16	16	>16	>16
Amikacin	4	>32	>32	8	4	1	4	≤2	8	16	>32
Cefiderocol	0.5	0.5	>8	0.012	0.25	0.12	0.5	2	4	1	1
Meropenem	8	8	16	4	2	16	1	4	8	>16	>16
Meropenem/vaborbactam	≤0.06	8	4	4	2	<0.06	1	4	2	>16	>16
Imipenem/relebactam	0.12	2	2	4	2	0.25	0.5	2	0.5	>8	8
Ceftazidime/avibactam	2	8	8	>16	2	2	16	>16	2	>16	>16
Eravacyclin	0.5	>0.5	>0.5	0.12	>0.5	0.25	>0.5	0.5	≤0.5	>0.5	>0.5
Ceftolozane/tazobactam	>8	4	>8	>8	2	>8	4	>8	>8	>8	>8
Piperacillin/tazobactam	>32	>32	>32	>32	>32	>32	>32	>32	>32	>32	16
Tobramycin	>4	4	>4		≤0.5		≤0.5	≤0.5	>4	2	4
Fosfomycin	≤16	64	>64		>64		>64	64	>64	>64	>64
Tigecyclin	1	>1	>1	0.25	>1	0.5	>1	1	≤0.5	>1	>1
Outcome	Cure	Cure	Cure	Death (Non infectious cause)	Cure	Cure	Cure	Suppressive antibiotic treatment	Death (Non infectious cause)	Cure	Cure

MIC: Minimal inhibitory concentration.

## Data Availability

All data are available upon request to the corresponding author.
